# Sleep traits, fat accumulation, and glycemic traits in relation to gastroesophageal reflux disease: A Mendelian randomization study

**DOI:** 10.3389/fnut.2023.1106769

**Published:** 2023-02-21

**Authors:** Xiaoyan Zhao, Rui Ding, Chengguo Su, Rensong Yue

**Affiliations:** ^1^Clinical Medical School, Chengdu University of Traditional Chinese Medicine, Chengdu, China; ^2^Department of Endocrinology, Hospital of Chengdu University of Traditional Chinese Medicine, Chengdu, China; ^3^Acupuncture and Tuina School, Chengdu University of Traditional Chinese Medicine, Chengdu, China

**Keywords:** Mendelian randomization, sleep, fat accumulation, type 2 diabetes, gastroesophageal reflux disease

## Abstract

**Background:**

Sleep traits, fat accumulation, and glycemic traits are associated with gastroesophageal reflux disease (GERD) in observational studies. However, whether their associations are causal remains unknown. We performed a Mendelian randomization (MR) study to determine these causal relationships.

**Methods:**

Independent genetic variants associated with insomnia, sleep duration, short sleep duration, body fat percentage, visceral adipose tissue (VAT) mass, type 2 diabetes, fasting glucose, and fasting insulin at the genome-wide significance level were selected as instrumental variables. Summary-level data for GERD were derived from a genome-wide association meta-analysis including 78,707 cases and 288,734 controls of European descent. Inverse variance weighted (IVW) was used for the main analysis, with weighted median and MR-Egger as complements to IVW. Sensitivity analyses were performed using Cochran’s *Q* test, MR-Egger intercept test, and leave-one-out analysis to estimate the stability of the results.

**Results:**

The MR study showed the causal relationships of genetically predicted insomnia (odds ratio [OR] = 1.306, 95% confidence interval [CI] 1.261 to 1.352; *p* = 2.24 × 10^−51^), short sleep duration (OR = 1.304, 95% CI: 1.147 to 1.483, *p* = 4.83 × 10^−5^), body fat percentage (OR = 1.793, 95% CI 1.496 to 2.149; *p* = 2.68 × 10^−10^), and visceral adipose tissue (OR = 2.090, 95% CI 1.963 to 2.225; *p* = 4.42 × 10^−117^) with the risk of GERD. There was little evidence for causal associations between genetically predicted glycemic traits and GERD. In multivariable analyses, genetically predicted VAT accumulation, insomnia, and decreased sleep duration were associated with an increased risk of GERD.

**Conclusion:**

This study suggests the possible roles of insomnia, short sleep, body fat percentage, and visceral adiposity in the development of GERD.

## 1. Introduction

Gastroesophageal reflux disease (GERD) is defined as a condition that develops when the reflux of stomach contents causes troublesome symptoms and/or complications (1). GERD is a highly prevalent disease, affecting approximately 13% of the worldwide population and 20% of the adult population in high-income countries (2, 3). GERD is linked to the increased risk of esophagitis, esophageal strictures, Barrett esophagus, and esophageal adenocarcinoma (2).

Sleep disturbance is commonly associated with GERD (4). Of 11,685 survey respondents with GERD, 88.9% experienced nighttime GERD symptoms, and 68.3% had sleep difficulties (5). Also, obesity increased the risk of GERD (6) and a causal association between body mass index (BMI) and GERD has been found in a Mendelian randomization study (7). However, BMI is a crude indicator of obesity because it does not account for body composition (8). Obesity is clinically defined based on the measurement of body fat percentage, subcutaneous adipose tissue, and visceral adipose tissue (VAT) (9). And VAT is considered unique pathogenic fat depots (10). In addition, diabetic patients suffer various complications, among which esophageal dysfunction are common (11). A meta-analysis suggested that patients with diabetes are at greater risk of GERD than those without diabetes (12). There is no evidence to demonstrate a causal relationship between fasting insulin, fasting glucose and GERD. Existing large-scale meta-analyses and observational studies have revealed several possible risk factors for GERD, including sleep disturbances (13, 14), excess adiposity (15–17), and diabetes mellitus (12). However, unobserved confounding factors, reverse causality, and other biases may affect these results in observational studies. Determining the causal relationship of sleep traits, fat accumulation, and glycemic traits with GERD is very important for the prevention and management of GERD.

Mendelian randomization (MR) is a credible and powerful method to investigate the causal relationship by using genetic variants associated with the specific exposures as instrumental variables (IVs) (18). The MR design can minimize the biases including residual confounding and reverse causality, because genetic variants are randomly allocated at conception (19). In this study, we performed a two-sample MR study to examine whether insomnia, sleep duration, short sleep duration, body fat percentage, VAT accumulation, type 2 diabetes mellitus (T2DM), fasting glucose, and fasting insulin were causally associated with the risk of GERD.

## 2. Methods

### 2.1. Study design

**Figure 1:**
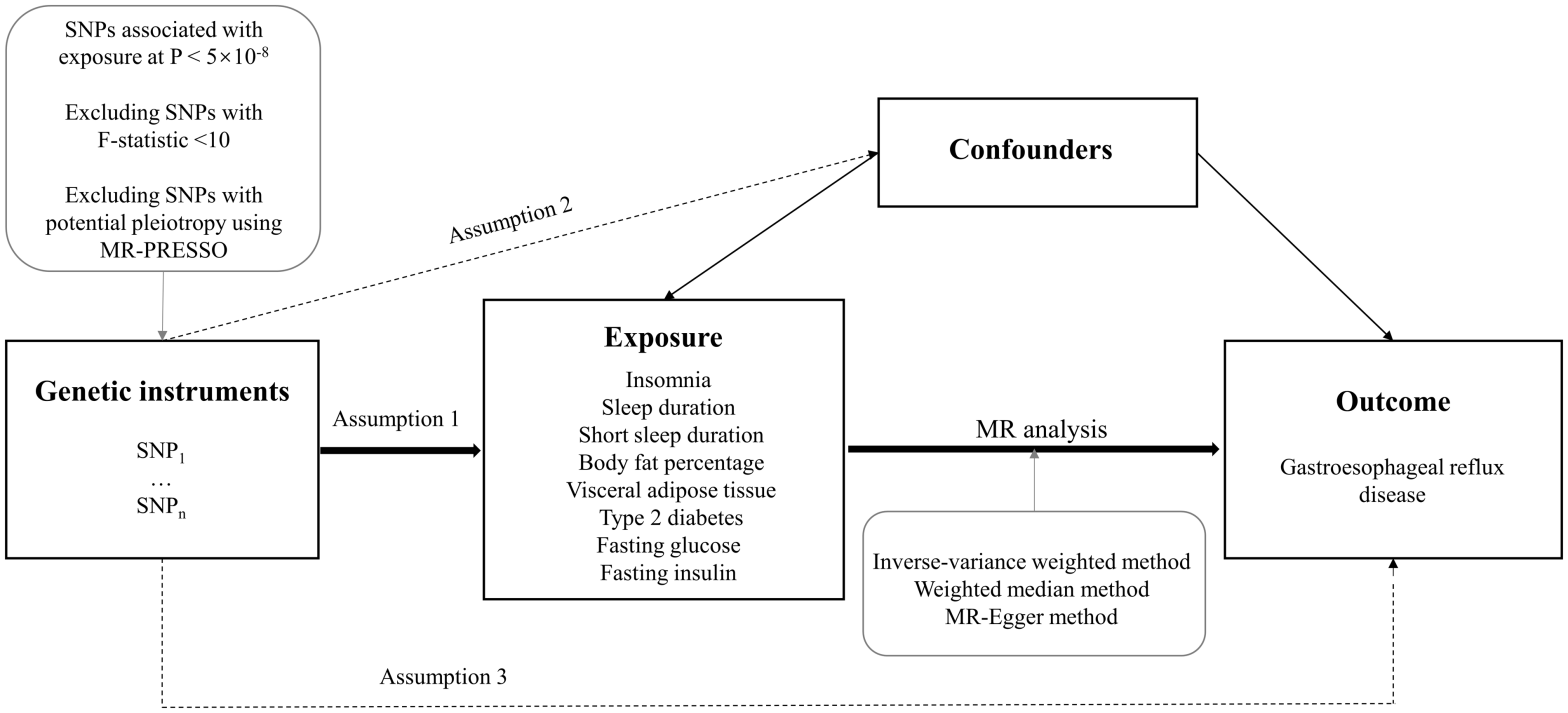
Study design overview. SNPs, single-nucleotide polymorphisms; MR, Mendelian randomization; MR-PRESSO, MR-pleiotropy residual sum and outlier.

The study design overview was shown in Figure 1. This study was based on summary-level data on measures of insomnia, sleep duration, short sleep duration, body fat percentage, visceral adipose tissue mass, type 2 diabetes, fasting glucose, fasting insulin, and GERD from published genome-wide association studies (GWASs). To obtain unbiased causal evaluations, the MR study needs to satisfy three assumptions: (1) the genetic variants are robustly associated with the exposure; (2) genetic variants are not associated with potential confounders; and (3) genetic variants affect the risk of the outcome only through the exposure. All analyses in our study were based on publicly available GWAS data, and no additional ethics approval was required.

### 2.2. Data sources for sleep traits, fat accumulation, and glycemic traits

Summary-level data for insomnia were obtained from a large meta-analysis of GWAS, including 1,331,010 European-ancestry individuals from UK Biobank (*N* = 386,533) and 23andMe (*N* = 944,477) (20). Insomnia complaints were measured using questionnaire data; an independent sample (the Netherlands Sleep Register), which provides similar question data and clinical interviews evaluating insomnia, was used to validate the specific questions, making them good proxies for insomnia (20). Genetic association data for sleep duration were obtained from a recently published GWAS of 446,118 European-ancestry participants (21). Participants were asked: “How many hours do you sleep in every 24 h?” This genome-wide association study identified 78 loci for self-reported sleep duration. Furthermore, the 78 loci were associated with accelerometer-derived sleep duration (*n* = 85,499) (21). Sleep duration was a continuous variable, and sleep duration <7 h was defined as short sleep, while sleep duration ≥9 h was considered long sleep (21). The number of single-nucleotide polymorphisms (SNPs) for long sleep duration was insufficient, which was under the risk of weak instrument bias. Therefore, long sleep duration was not included in our study.

IVs for body fat percentage (BF%) were available from a genome-wide association meta-analysis of 100,716 individuals (22). Of these, summary statistics from 65,831 individuals of European ancestry were included in our study (22). BF% was measured either with dual-energy *X*-ray absorptiometry (DEXA) or bioimpedance analysis (BIA), as described in detail before (23). The summary data for VAT accumulation were derived from a large-scale GWAS including 325,153 participants (24). This GWAS study constructed two sub-cohorts to calculate VAT mass: a VAT-training dataset with VAT mass estimated by DEXA, to which the prediction models were calibrated; and a VAT-application dataset, in which VAT mass was determined by the calibrated prediction models (24).

We extracted summarized data for T2DM based on 659,361 participants, fasting glucose based on 200,622 participants, and fasting insulin based on 151,013 participants from the MRC IEU Open GWAS database (ID: ebi-a-GCST006867 for T2DM, ebi-a-GCST90002232 for fasting glucose, ebi-a-GCST90002238 for fasting insulin) (25–28).

### 2.3. Data source for gastroesophageal reflux disease

IVs for GERD were obtained from a recent GWAS meta-analysis of the QSKIN study and UK Biobank study including 78,707 cases and 288,734 controls of European descent (29). The QSkin cohort (30) is a population-based cohort study to investigate risk factors for skin cancers and other complex diseases in Queensland, Australia. The UK Biobank (31) is a large-scale population-based cohort consisting of over 500,000 participants aged 40–69 years recruited from the United Kingdom. GERD cases were defined based on a combination of self-reported GERD symptoms, international classification of diseases diagnosis, and the use of GERD-related medication (29). Individuals that did not have any history or occurring conditions of disorders in the upper digestive system were defined as controls. In the multi-trait genetic association analysis, 88 loci associated with GERD were identified (29). The GWASs details in the MR study were summarized in Table 1.

**Table 1:** The data information involved in univariate MR study.

Phenotype	Author, published year	Sample size	No. of cases (Binary trait)	PMID
Insomnia	Jansen PR et al., 2019 (20)	1,331,010	288,557	30,804,565
Sleep duration	Dashti HS et al., 2019 (21)	446,118	NA	30,846,698
Short sleep duration	Dashti HS et al., 2019 (21)	411,934	106,192	30,846,698
Body fat percentage	Lu YC et al., 2016 (22)	65,831	NA	26,833,246
Visceral adipose tissue	Karlsson T et al., 2019 (24)	325,153	NA	31,501,611
Type 2 diabetes	Xue A et al., 2018 (26)	659,316	62,892	30,054,458
Fasting glucose	Chen J et al., 2021 (25)	200,622	NA	34,059,833
Fasting insulin	Chen J et al., 2021 (25)	151,013	NA	34,059,833
Gastroesophageal reflux disease	Ong JS et al., 2022 (29)	367,441	78,707	34,187,846

### 2.4. Selection of genetic instruments

To filter eligible instrumental variables, we performed rigorous filtering steps before MR analysis. First, we selected genetic instrumental variables that were significantly associated with the exposures at a genome-wide significance level of *p* < 5 × 10^−8^. Second, the PLINK clumping algorithm (*r*^2^ = 0.001 and window size = 10,000 kb) was performed to evaluate the linkage disequilibrium. Third, we selected the SNPs with F statistic >10. IVs with F statistic less than 10 were considered weak genetic instruments (32). Fourth, ambiguous and palindromic SNPs derived from harmonizing processes were excluded. Finally, SNPs with potential pleiotropy were removed using the MR-pleiotropy residual sum and outlier (MR-PRESSO) analysis. MR-PRESSO analysis could correct horizontal pleiotropy *via* outlier removal (33).

### 2.5. Mendelian randomization estimates

Several MR approaches, including inverse variance weighted (IVW), weighted median (WM), and MR-Egger were performed to evaluate the causal association of exposure with outcome. IVW was used as the main statistical method (the random-effects model for the exposure constructed by ≥ 3 SNPs) (7), with WM and MR-Egger methods as supplements to IVW. IVW analysis provides the most precise results when all selected SNPs are valid IVs (34). The WM method can provide a consistent estimate even when up to 50% of the information comes from invalid IVs (35). MR-Egger analysis can be used for detecting violations of the instrumental variable assumptions, but causal estimates from the MR-Egger may be biased and have inflated type 1 error rates (36). For quality control, the intercept term derived from the MR-Egger regression was used to evaluate horizontal pleiotropy. Cochran’s *Q*-test was performed to assess heterogeneity. We also performed leave-one-out analyses by excluding each SNP to evaluate whether causal estimates are driven by a single SNP.

### 2.6. Multivariable Mendelian randomization

Obesity is a potential confounder affecting the risk of GERD (29, 37). Therefore, we performed a multivariable IVW MR analysis to confirm the direct effects of sleep traits and fat accumulation after adjusting for body mass index (BMI). Genetic variables on BMI were obtained from the Genetic Investigation of Anthropometric Traits (GIANT) consortium (38). Detailed information on data source for the multivariable MR study was displayed in Supplementary Table S1.

### 2.7. Statistical analysis

All analyses were performed using the TwoSampleMR package (version 0.5.6) in the R software (version 4.2.1). The MR estimates were shown as odds ratios (OR) with corresponding 95% confidence intervals (CI). To account for multiple testing, we used the Bonferroni correction to adjust the thresholds for significance. Associations with *p* value < 0.006 (0.05/8 exposures) were regarded as significant associations, and associations with *p* value ≥ 0.006 and < 0.05 were considered suggestive associations.

## 3. Results

### 3.1. Genetic instruments

In total, there were 105 SNPs as instrumental variables for insomnia, 36 SNPs for sleep duration, 14 SNPs for short sleep duration, 7 SNPs for body fat percentage, 136 SNPs for VAT, 100 SNPs for T2DM, 44 SNPs for fasting glucose, and 16 SNPs for fasting insulin. The SNP instruments for the causal relationship between these traits and GERD were detailed in Supplementary Tables S2–S9.

### 3.2. Causal associations between sleep traits and gastroesophageal reflux disease

**Figure 2:**
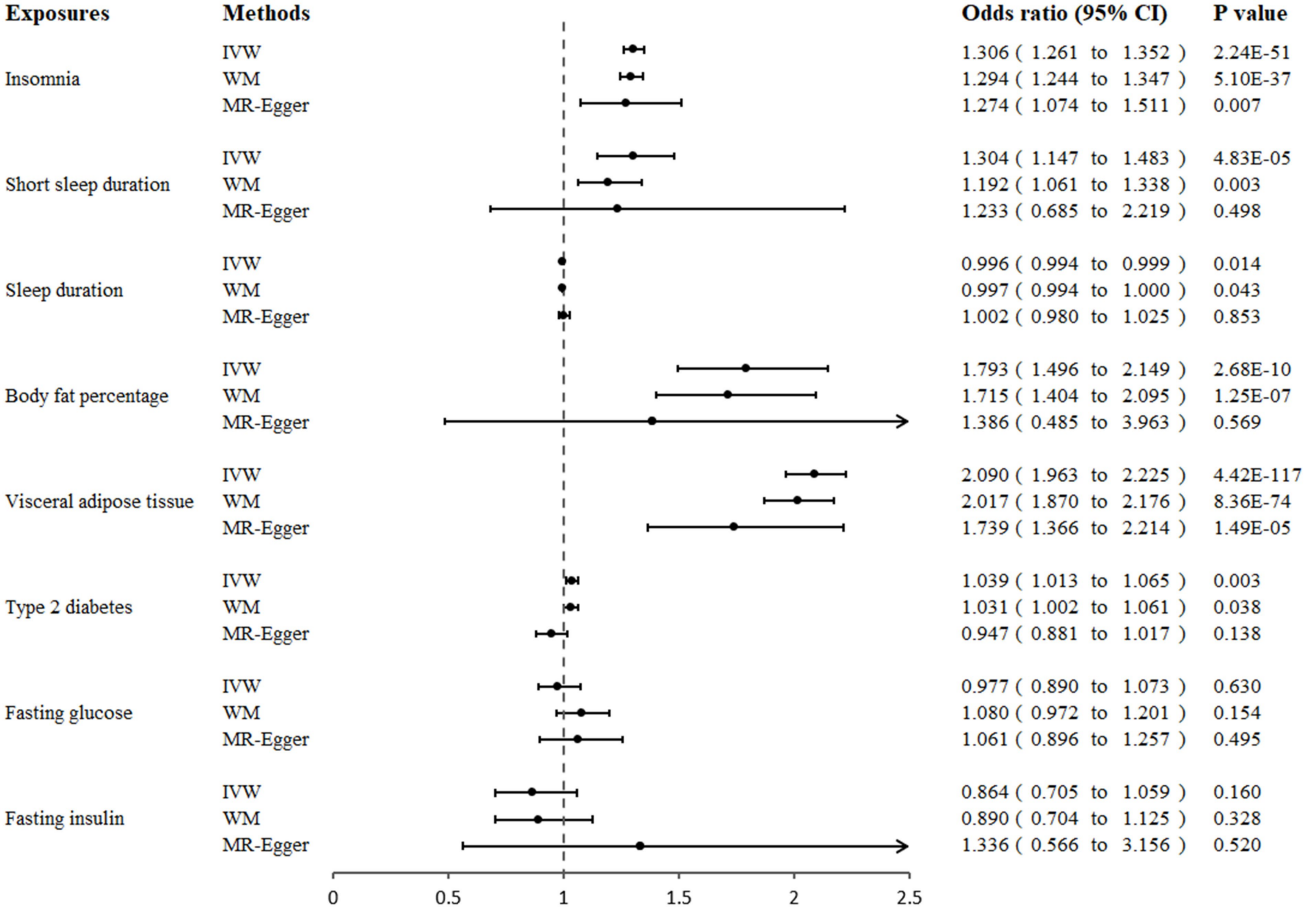
Association of genetically proxied sleep traits, fat accumulation, and glycemic traits with gastroesophageal reflux disease in Mendelian randomization analyses. MR, Mendelian randomization; IVW, inverse-variance weighted; WM, weighted median.

**Figure 3:**
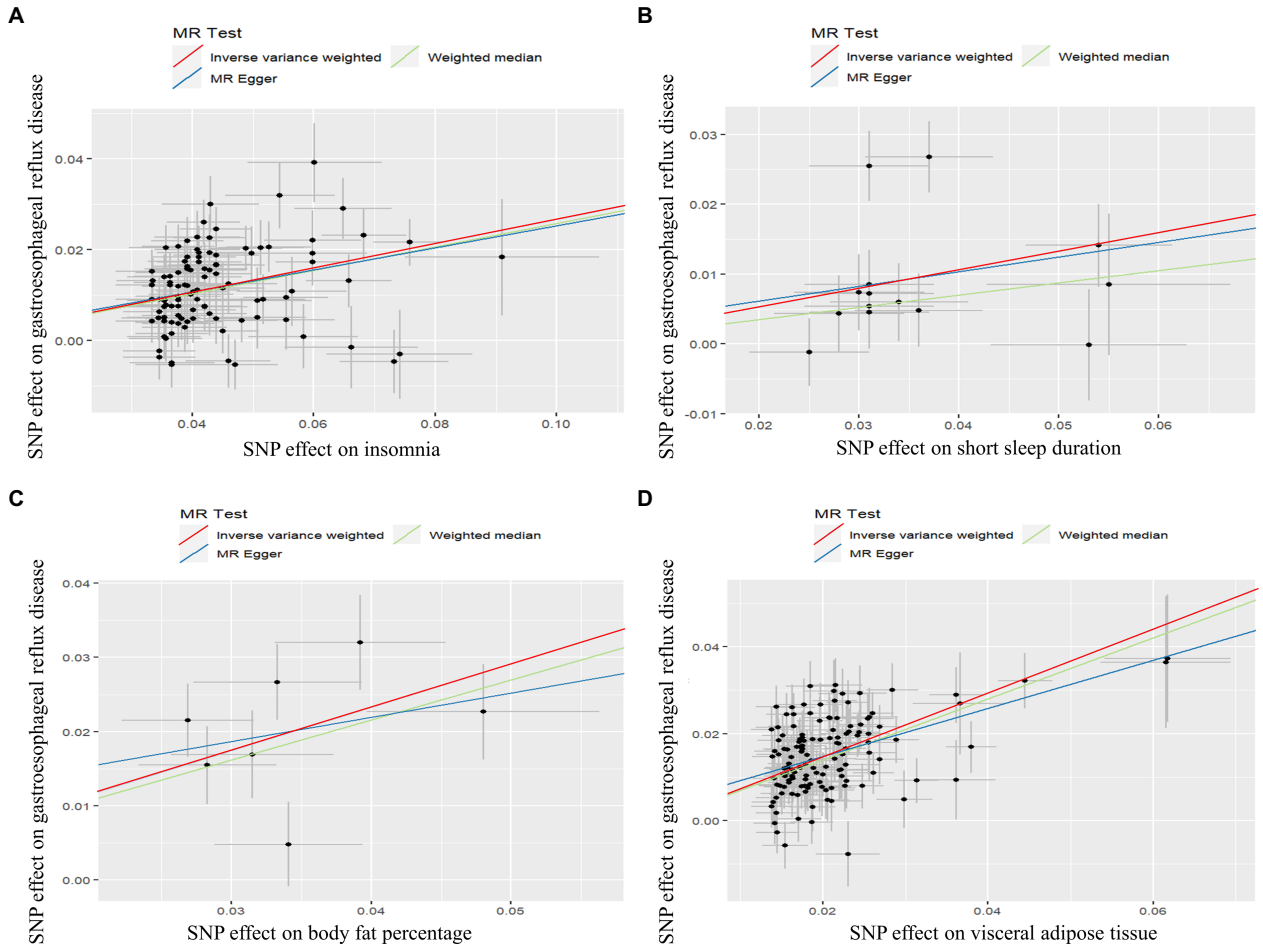
Scatter plot of Mendelian randomization analysis for the associations of insomnia **(A)**, short sleep duration **(B)**, body fat percentage **(C)**, and visceral adipose tissue **(D)** with the risk of gastroesophageal reflux disease.

Genetically predicted insomnia was significantly associated with an increased risk of GERD in both the IVW analysis (OR = 1.306, 95% CI 1.261 to 1.352; *p* = 2.24 × 10^−51^) and the WM analysis (OR = 1.294, 95% CI 1.244 to 1.347; *p* = 5.10 × 10^−37^). And a suggestive association was presented in the MR-Egger analysis (OR = 1.274, 95% CI 1.074 to 1.151; *p* = 0.007; Figures 2, 3A). Moreover, IVW and WM analyses demonstrated that short sleep duration was significantly associated with the risk of GERD (OR = 1.304, 95% CI: 1.147 to 1.483, *p* = 4.83 × 10^−5^; OR = 1.192, 95% CI: 1.061 to 1.338, *p* = 0.003), whereas MR-Egger presented a consistent direction but nonsignificant result (Figures 2, 3B). In addition, the results from IVW suggested that there was a suggestive association between sleep duration and GERD (OR = 0.996, 95% CI 0.994 to 0.999; *p* = 0.014), whereas the causal evaluations from MR-Egger showed an inconsistent direction (Figure 2).

### 3.3. Causal associations between body fat percentage, visceral adipose tissue, and gastroesophageal reflux disease

A significant positive correlation between genetically proxied body fat percentage and GERD risk was detected using IVW analysis (OR = 1.793, 95% CI 1.496 to 2.149; *p* = 2.68 × 10^−10^) and weighted median (OR = 1.715, 95% CI 1.404 to 2.095; *p* = 1.25 × 10^−7^), and MR-Egger analysis showed a similar causal estimate, although the association was not statistically significant (Figures 2, 3C). In addition, a significant positive causality between VAT accumulation and GERD risk was presented in the IVW analysis (OR = 2.090, 95% CI 1.963 to 2.225; *p* = 4.42 × 10^−117^), weighted median (OR = 2.017, 95% CI 1.870 to 2.176; *p* = 8.36 × 10^−74^), and MR-egger (OR = 1.739, 95% CI 1.366 to 2.214; *p* = 1.49 × 10^−5^; Figures 2, 3D).

### 3.4. Causal associations between T2DM, fasting glucose, fasting insulin, and gastroesophageal reflux disease

In our primary analysis, genetically predicted T2DM was associated with an increased risk of GERD (OR = 1.039, 95% CI 1.013 to 1.065; *p* = 0.003). A suggestive association was also shown in the weighted median analysis (OR = 1.031, 95% CI 1.002 to 1.061; *p* = 0.038). However, the causal inference from MR-Egger showed an inconsistent direction (OR = 0.947, 95% CI 0.881 to 1.017; *p* = 0.138). Additionally, we did not observe any association of genetically predicted fasting glucose or fasting insulin with GERD risk in three MR analyses (Figure 2).

### 3.5. Sensitivity analyses

Cochran’s *Q* test and MR-Egger intercept test were performed to evaluate the robustness of these causal estimates (Table 2). Although heterogeneity was observed for some results in the Cochran’s *Q* test, it did not invalidate the MR results from the random-effect IVW method, which might balance the pooled heterogeneity (39). Horizontal pleiotropy was detected in the MR-Egger intercept test of T2DM (*P* for intercept < 0.05). However, the *p*-values of other evaluations were all > 0.05, indicating no horizontal pleiotropy bias was introduced into the MR estimates in the context of heterogeneity (39). Leave-one-out analyses demonstrated that the estimates in our study were not biased by any single SNP.

**Table 2:** Sensitivity analysis of the associations between sleep traits, fat accumulation, glycemic traits and gastroesophageal reflux disease.

Outcome	Exposure	Cochran *Q* test		MR-Egger	
		*Q* value	*P*	Intercept	*P*
Gastroesophageal reflux disease	Insomnia	220.834	2.06E-10	0.001	0.773
	Short sleep duration	31.344	0.003	0.002	0.851
	Sleep duration	80.442	3.04E-05	0.001	0.822
	Body fat percentage	13.313	0.038	0.009	0.646
	Visceral adipose tissue	258.186	9.33E-10	0.004	0.125
	Type 2 diabetes	225.847	6.59E-12	0.007	0.009
	Fasting glucose	81.392	0.0004	−0.002	0.259
	Fasting insulin	27.696	0.024	−0.008	0.324

### 3.6. Multivariable Mendelian randomization

Considering obesity plays an important role in the pathogenesis of GERD, we then conducted an IVW-based multivariable MR to estimate the effects of insomnia, sleep duration, body fat percentage, and VAT on GERD accounting for the confounding effect from BMI. In multivariable IVW analysis, after adjusting for BMI, the effects of insomnia (OR = 3.225, 95% CI 2.347 to 4.431; *p* = 5.07 × 10^−13^), sleep duration (OR = 0.642, 95% CI 0.508 to 0.813; *p* = 0.0002), and VAT (OR = 1.223, 95% CI 1.025 to 1.459; *p* = 0.026) on GERD did not alter substantially, respectively. However, the association between body fat percentage and risk of GERD was not significant after adjusting for BMI (Table 3).

**Table 3:** Multivariable Mendelian randomization results.

Exposure	Adjustment	MR method	*P* value	OR (95% CI)
Insomnia	BMI	IVW	5.07E-13	3.225 (2.347, 4.431)
Sleep duration	BMI	IVW	0.0002	0.642 (0.508, 0.813)
Body fat percentage	BMI	IVW	0.127	1.254 (0.938, 1.676)
Visceral adipose tissue	BMI	IVW	0.026	1.223 (1.025, 1.459)

## 4. Discussion

In the current study, MR was performed to investigate the causal relationships of insomnia, sleep duration, short sleep duration, body fat percentage, VAT accumulation, T2DM, fasting glucose, and fasting insulin with GERD. We found some evidence that genetically predicted insomnia, short sleep duration, body fat percentage, and VAT accumulation were associated with an increased risk of GERD. Obesity is a known GERD risk factor. Our study showed that VAT accumulation, insomnia, and decreased sleep duration were associated with an increased risk of GERD after adjusting for BMI.

Sleep disturbances were associated with GERD. A total of 61.7% of 33,391 French patients with GERD had regular GERD-related sleep disturbances (40). A cohort study including 3,813 GERD cases and 15,252 controls indicated that GERD might increase the risk of sleep disorders (41). A large population-based study suggested that the association between sleep problems and GERD might be bidirectional (42). Among the many lifestyle factors, poor quality of sleep is a strong risk factor for GERD (13). Previous studies observed that persistent insufficient and/or short sleep increased the risk of GERD (13, 14). A prospective study including 2,316 adults showed that insomnia was associated with the risk of GERD (43). Consistent with previous studies, our MR study confirmed the causal association of insomnia and short sleep with the increased risk of GERD. The study suggests that treatments to improve sleep quality may decrease GERD symptoms in patients with GERD. Poor sleep quality due to sleep fragmentation and sleep deprivation from awakenings is related to GERD symptoms (44). Several potential mechanisms may contribute to the causal relationship between sleep and GERD. A study in healthy adults has shown a hyperalgesic effect associated with sleep deprivation and an analgesic effect associated with slow-wave sleep recovery (45) and sleep deprivation may cause esophageal hyperalgesia evidenced by the acid perfusion testing, which provided an underlying mechanism for the GERD symptoms in patients with poor sleep quality (46). In addition, we considered the interpretation of the results in terms of psychological factors. Insomnia is a prevalent mental disorder (20). Previous studies have shown a bidirectional association between sleep disorders and depression (47, 48). A study indicated that depressive symptoms and poor sleep quality are associated with the presence of GERD (49). Psychopathology plays a role in GERD pathogenesis (50). A study suggested that psychological symptomatology, mood and anxiety disorders are positively associated with GERD symptoms (50). Another study observed the associations between anxiety, poor sleep quality and GERD (51). In GERD patients, there was a strong relationship between psychological stress (anxiety and depression) and sleep disturbances (52). Therefore, sleep may affect GERD through factors such as depression.

Overweight and obesity were correlated with an increased risk of GERD (6, 15). A meta-analysis including 18,346 patients with GERD demonstrated a positive relationship between increasing BMI and GERD risk (6). Another meta-analysis showed that obesity was associated with a significant increase in the risk for GERD and its complications (erosive esophagitis, and esophageal adenocarcinoma) (15). And the risk of these diseases seems to progressively increase with weight gain (15). Weight loss was dose-dependently correlated with both a reduction of GERD symptoms and an increase of treatment success with antireflux medication (16). BMI is calculated with weight and height, which does not consider body composition such as body fat mass and VAT mass. Body fat distribution, especially the abdominal adiposity (such as visceral adiposity), is an important factor in the association of obesity with GERD, and it is more strongly associated with GERD than BMI (53). Our study showed that genetically predicted body fat percentage and VAT mass were positively associated with the risk of GERD. In multivariable IVW analysis, the association between body fat percentage and GERD risk was not significant after adjusting for BMI, suggesting that this association could be affected by BMI. In addition, a positive association between VAT and GERD after adjusting for BMI was observed, which confirmed the robustness of the result. The accumulation of VAT is more harmful than the accumulation of adipose tissue at other locations (54, 55). Several potential mechanisms may explain the association, including increased intra-abdominal pressures, delayed gastric emptying, low esophageal sphincter abnormalities, and increased frequency of transient sphincter relaxation (15, 37). And visceral adipose tissue, which is metabolically active, secretes adipokines and inflammatory cytokines that may predispose to GERD and its complications (37).

Gastrointestinal symptoms are common in patients with diabetes. A meta-analysis involving 9,067 cases and 81,968 controls suggested that individuals with diabetes were at greater risk of GERD than those without diabetes (overall OR = 1.61) (12). Our main analysis showed the causal nature of the positive association between T2DM and GERD, but three MR analyses presented an inconsistent result. In addition, our MR estimates could not provide any association of genetically predicted fasting glucose or fasting insulin with GERD. Inconsistent with the previous studies, the correlation between glycemic traits and GERD could not be determined in our study. But it’s worth noting that diabetic upstream factors (e.g., obesity), duration of diabetes, and diabetic autonomic neuropathy were associated with gastrointestinal symptoms (12, 56, 57). It has been reported that patients with diabetic complications are more likely to report reflux symptoms, and the quality of diabetic control and use of oral hypoglycemic agents may influence the incidence of GERD (58). Studies have reported on the association between diabetes and esophageal dysfunction; however, no consensus has been reached. The mechanism for this association needs to be further explored.

Our study has several important strengths. First, MR is an analytic approach using genetic variants as IVs to explore the causal association between exposure and outcome. MR design diminished unobserved confounding and reverse causality that are common in observational studies. Second, several MR methods and sensitivity analyses were performed to ensure the stability of the results. Finally, our study strengthened the requirement for consistent beta direction in all MR analyses (39). IVW has higher statistical power than other MR analyses, especially MR-Egger (59). Therefore, it is not surprising that the causal estimates derived from the MR-Egger may have nonsignificant *p*-values and wider confidence compared to IVW estimates in our study (39).

Our study also has several limitations. First, most participants in the datasets were of European ancestry, which may limit the generalizability of the findings to other ethnic groups. Second, it is difficult to completely exclude pleiotropy in MR analyses. Horizontal pleiotropy affects the stability of MR results, but vertical pleiotropy where exposure acts on outcome through other factors with the same causal pathway is acceptable. Importantly, the MR-Egger regression method detected no evidence of horizontal pleiotropy for important causal evaluations in our study. Third, self-reported sleep data may introduce potential bias into the study. Despite limitations of imprecision in self-report, the GWAS study observed largely consistent effects of the 78 signals for self-reported sleep duration with accelerometer-estimated sleep duration (21). Finally, GERD cases are defined using various sources (self-reported GERD, medication use, and clinical diagnosis), which may introduce outcome misclassification. However, the GERD genome-wide association study found very high-genetic correlations (*r*_g_ > 0.9) between the different GERD phenotypes (29), indicating a good validity of GERD data. Thus, the bias caused by GERD data is not a major issue.

## 5. Conclusion

Our study suggested that genetically predicted insomnia, short sleep duration, and visceral adipose tissue accumulation were correlated with an increased risk of GERD. Thus, improving sleep quality and reducing visceral adiposity may be potential intervention targets for preventing GERD.

## Data availability statement

The original contributions presented in the study are included in the article/Supplementary material, further inquiries can be directed to the corresponding authors.

## Author contributions

XZ, CS, RD, and RY designed the manuscript. XZ and CS are responsible for the statistical analyses and manuscript writing. All authors contributed to the article and approved the submitted version.

## Funding

This project was supported by Natural Science Foundation of Sichuan Province (2022NSFSC0853).

## Conflict of interest

The authors declare that the research was conducted in the absence of any commercial or financial relationships that could be construed as a potential conflict of interest.

## Publisher’s note

All claims expressed in this article are solely those of the authors and do not necessarily represent those of their affiliated organizations, or those of the publisher, the editors and the reviewers. Any product that may be evaluated in this article, or claim that may be made by its manufacturer, is not guaranteed or endorsed by the publisher.
